# Engagement of Components of DNA-Break Repair Complex and NFκB in Hsp70A1A Transcription Upregulation by Heat Shock

**DOI:** 10.1371/journal.pone.0168165

**Published:** 2017-01-18

**Authors:** Joyita Hazra, Pooja Mukherjee, Asif Ali, Soumita Poddar, Mahadeb Pal

**Affiliations:** 1 Division of Molecular Medicine, Bose Institute, P1/12, CIT Scheme VIIM, Kolkata, India; 2 Bioinformatics Center, Bose Institute, P1/12, CIT Scheme VIIM, Kolkata, India; Southern Illinois University School of Medicine, UNITED STATES

## Abstract

An involvement of components of DNA-break repair (DBR) complex including DNA-dependent protein kinase (DNA-PK) and poly-ADP-ribose polymerase 1 (PARP-1) in transcription regulation in response to distinct cellular signalling has been revealed by different laboratories. Here, we explored the involvement of DNA-PK and PARP-1 in the heat shock induced transcription of Hsp70A1A. We find that inhibition of both the catalytic subunit of DNA-PK (DNA-PKc), and Ku70, a regulatory subunit of DNA-PK holo-enzyme compromises transcription of Hsp70A1A under heat shock treatment. In immunoprecipitation based experiments we find that Ku70 or DNA-PK holoenzyme associates with NFκB. This NFκB associated complex also carries PARP-1. Downregulation of both NFκB and PARP-1 compromises Hsp70A1A transcription induced by heat shock treatment. Alteration of three bases by site directed mutagenesis within the consensus κB sequence motif identified on the promoter affected inducibility of Hsp70A1A transcription by heat shock treatment. These results suggest that NFκB engaged with the κB motif on the promoter cooperates in Hsp70A1A activation under heat shock in human cells as part of a DBR complex including DNA-PK and PARP-1.

## Introduction

Cells often elevate the level of their protein chaperones in response to stressful internal and external environment to maintain homeostasis. Hsp70A1A and Hsp70A1B are among the most common and important players in the inducible protein chaperone family [1]. The genes encoding these proteins are located 12 base pair apart on the short arm of chromosome 6 (6p21.3) between the major histocompatibility class III and the TNF loci [2,3,4]. Although these two genes encode very similar proteins of 641 amino acids except differing in two amino acids at positions 110 and 499, they differ in their regulatory regions such as the promoters and the UTRs [3,5]. Both Hsp70A1A and Hsp70A1B proteins are expressed in almost all cell types with the former expressing relatively in a higher level.

Functionally mouse deficient in Hsp70 genes is susceptible to cerebral ischemia, TNF-induced lethal inflammatory shock, osmotic stress, UV irradiation and pancreatitis [5,6,7,8,9]. Overexpression of Hsp70 (Hsp70A1A and Hsp70A1B) genes provides resistance to TNF-induced apoptosis [10,11]. Constant upregulation of Hsp70 was associated with oncogenesis and resistance to chemotherapy [12] while a decrease in Hsp70 level has been correlated with increased protein misfolding, and aggregation associated neurodegenerative diseases [13,14]. In malignantly transformed cells Hsp70 supports rapid proliferation, resistance to stress-induced apoptosis, cytostatic drugs, and radiation therapy and suppresses cellular senescence [15].

While the involvement of Hsp70 protein in various diseases including cancer is well correlated, transcription regulation of Hsp70 gene is relatively not well understood. The gene is upregulated in various stressful conditions such as exposure to elevated temperature, ROS, and heavy metal through activation of heat shock factor 1 (HSF1). HSF1, a transcription activator executes it function through binding to heat shock elements (HSE) motif present in multiple copies on its target promoters [16]. In a healthy cellular condition, a basal level Hsp70 expression can be attributed to the presence of binding of several transcription factors on its promoter of Hsp70 such as TATA-, CCAAT-, and NF-Y motif binding proteins [17]. Recently, an involvement of several transcription factors such as NFκB along with NF-Y, and CREB in the basal expression of the Hsp70 gene in mice has been demonstrated. Sequence alignment of promoters of mouse and human Hsp70A1A genes revealed only 51% similarity [18]. It is not investigated how if these transcription factors extend any cooperation with heat shock factor 1 (HSF1) function under a stressor such as heat shock.

Dependence of human Hsp70A1A on transcription factors like NFκB under heat shock condition has not been tested. Nuclear factor κB (NFκB) is a well studied transcription factor for its crucial roles in many cellular processes such as inflammation, cell growth, proliferation, and apoptosis. NFκB in cells is also responsive to genotoxic and oxidative stressors. The NFκB family contains five distinct classes of proteins named- RelA (p65), RelB, c-Rel, p100 (precursor of p50) and p105 (precursor of p52). The NFκB group of proteins normally function as a dimer with the most predominant being the p65/p50 heterodimer present in almost all cells in relatively an abundant level [19]. Normally, NFκB is sequestered in the cytoplasm in a complex with IκB. The NFκB translocates to the nucleus as IκB is marked by IKK through phosphorylation and is degraded by proteasome in response to an activation signal [20].

HSF1 regulates heat shock response through binding to its recognition sequence heat shock element (HSE) on its target gene promoter. Evidence suggests that posttranslational modification such as phosphorylation, sumoylation, acetylation of HSF1 regulates its function in response to heat shock. Phosphorylation of HSF1 was correlated both with its activation as well as inactivation. Indeed, several kinases such as calcium/calmodulin dependent kinase, glycogen synthase kinase, and mitogen activated protein kinase (MAPK)/ERK have been implicated either in activation or repression of HSF1 activity [21,22]. However, an involvement of a kinase such as DNA-PK in the heat shock induced transcription of Hsp70 has not been adequately addressed.

DNA-PK is a well studied player in the repair of DNA double stranded break induced by various DNA damaging agents including reactive oxygen species. DNA-PK holoenzyme is composed of 450 kDa catalytic subunit and two regulatory DNA-binding proteins called Ku protein (Ku70- and Ku86 kDa). DNA-PK is a serine threonine kinase implicated in regulation of activity of several transcription factors and DNA binding proteins such as Ku proteins through phosphorylation. The importance of DNA-PK in the signal induced transcription in response to various stimuli has been demonstrated. Interestingly, components of DNA-break/repair complex such as PARP-1 and Topoisomerae IIβ (TopoIIβ) were found to be in a complex with DNA-PK [23,24,25,26,27]. PARP-1 enzyme has been implicated in DNA repair and transcription, and was reported to be in a DNA-PK holoenzyme associated complex in the cell especially after DNA-damage [28]. It is a nuclear enzyme that modifies the target proteins by addition of negatively charged ribose moieties from nicotinamide nucleotide diphosphate (NAD) [29]. In fruit fly PARP-1 was shown to be required with heat shock factor (HSF) for the activation of the Hsp70 after heat shock treatment [30,31]. TopoIIβ, a component of DNA-repair machinery is known to help relax the chromatin structure by releasing its topological constrains. TopoIIβ was shown to create transient nucleosome specific DNA double stranded break in promoter nearby region to facilitate stimulus induced transcription [25,32].

Here we show that inhibition of DNA-PK and PARP-1 compromises Hsp70A1A transcription in cells in response to heat shock treatment. Interestingly, we find that these two proteins associate with NFκB in heat shock dependent manner. A downregulation of NFκB affects Hsp70A1A promoter activity under heat shock. Nucleotide sequence analysis identified a NFκB recognition motif on Hsp70A1A promoter. Alteration of three bases in that recognition motif compromises inducibility of Hsp70A1A gene upon heat shock.

## Materials and Methods

### Cell Lines, culture and maintenance

Human cervical cancer cell (HeLa), human colon carcinoma (HCT116), human fibrosarcoma (HT1080), human embryonic kidney cell (HEK293) originally purchased from ATCC were a kind gift from Dr. Andrei Gudkov, Roswell Park Cancer Institute (RPCI), Buffalo, NY. Cells were cultured in 37^°^C in a humidified atmosphere containing 5% CO_2_ in DMEM (Gibco) supplemented with 1 mM L-glutamine, 10% fetal bovine serum, 50 μg/ml penicillin, 50 μg/ml streptomycin and 2.5 μg/ml amphotericin B and non essential amino acids. Cells were grown in different sizes of cell culture plates to not more than 80–90% confluency.

### Plasmids, chemicals, and antibodies

FLAG-Ku70 plasmid was obtained from Shigemi Matsuyama, Case Western Reserve University, Cleveland as a kind gift. FLAG-p65 Plasmid was a kind gift from Katerina Gurova RPCI, Buffalo, NY. Inhibitor of DNA-PK, IC86621; anti-FLAG antibody and M2 affinity- and protein A/G beads were procured from Sigma, USA. DNA-PKc, p65/RelA and PARP-1 antibodies were purchased form Cell signaling Technology, USA. DNA-PKc antibody was also obtained from Kamiya Medical, USA. The anti-β-actin monoclonal antibody was obtained from Abcam, USA. siRNAs were bought from either Eurogenetic or Santacruz biotech, USA. The anti-his_6_ antibody was purchased from Biobhatarti life sciences, India. Andrographolide purified (>95% purity) from leaves of *Andrographis paniculata* was a kind gift from Vinod Kumar Nelson.

### Small interfering RNA (siRNA) treatment, RNA isolation, semiquatitative RT-PCR and RT-q PCR

Small interfering RNAs (siRNAs) were introduced in desired cells line through transfection using Lipofectamine 2000 (Invitrogen). Cells were lysed immediately, or after 45 min recovery following heat shock for 30 min. Usually cDNA was prepared with 1 μg of the total RNA using iScript cDNA synthesis kit according to the manufacturer’s protocol (BioRad). The efficacy of knockdown of mRNA level was estimated at 72 or 96 hour post transfection by semi-quantitative reverse transcriptase PCR (RT-PCR) or RT-qPCR using Taq polymerase with suitable cycles of amplification. As appropriate 25 to 35 cycles of reaction were used for PCR amplification of template. The level of β-actin or GAPDH mRNA was used for normalization or as loading control as appropriate. The following cDNA-specific primer pairs were used: DNA-PKc-forward 5’- CCAGCCCTGGACCTTCTTA-3’ and reverse 5’-CGGAACAGGTTTTCTGCATT-3’; Hsp70A1A-forward 5’-GCTGCGACAGTCCACTACCT-3’ and reverse 5’-TGCCGGTTCCCTGCTCTCTG -3’; Ku70-forward 5’-AGGATCATGCTGTTCACCAA-3’ and reverse 5’-CCAGGTTTCTTCAGGTGCAT-3’; p65/RelA-forward 5’-AATGGCTCGTCTGTAGTGC-3’ and reverse 5’-TGCTCAATGATCTCCACATAGG -3’; PARP-1-forward 5’-CGGGATTTCATCTGGTGTG -3’ and reverse 5’-AATTACCACAGGGAGGTCTT -3’; GAPDH-forward 5’-CGACCACTTTGTCAAGCTCA-3’ and reverse 5’-TTCCTCTTGTGCTCTTGCTG-3’; and β-actin-forward 5’-GCCGTCTTCCCCTCCATCGT-3’ and reverse 5’-CCTCGGTCAGCAGCACGGGG -3’; Rluc-forward 5’-TGGAGCCATTCAAGGAGAAG-3’ and reverse 5’- CGAAGGTAGGCGTTGTAGTT-3’, and GFP-forward 5’-GCGTGGAGGAGAACTGC -3’ and reverse 5’- GGATGATCTTCTCGCAGG-3’. The qPCR reaction was performed using sybr green (hot jumpstart sybr green, Sigma) according to the manufactures protocol on a 7500 Fast Real-time PCR system (Applied Biosystems) using GAPDH or β-actin as an internal control. The primers and dNTPs in the reactions were always kept in excess.

### Preparation of lentiviral stocks and construction of stable shDNA-PKc cell line

pTRIPZ-shDNA-PKc construct (a TET-ON system, obtained from Open Biosystems) carries a tetracycline inducible DNA-PKcshRNA adjacent to a constitutively active red fluorescent protein and puromycin selectable marker. Lentiviral particles as prepared with pTRIPZ-shDNA-PKc plasmid DNA in HEK293FT cells using second generation packaging plasmids as described elsewhere except that calcium phosphate was used for transfection [33]. Cells infected with a 2-fold excess of viral particles were subjected to puromycin (1 μg/ml) selection next day (after 24 h post infection). Selection on puromycin was carried out for two weeks. The expression of shDNA-PKc was induced by treating the cells with 1 μg/ml of doxycycline (TET-ON) for 72 h.

### Construction of reporter plasmids with DNA containing different portions of Hsp70A1A promoter

Different deleted versions of Hsp70A1A promoter were cloned in pGL4.79 at XhoI/HindIII sites. Hsp70A1A promoter regions were isolated by PCR using genomic DNA as the template using specific sets of primers. For cloning region between -450 and +91, and -750 and +330 the primers -450-forward 5’ATAT**CTCGAG**GGTCAGAGACAGTATCTCCA 3’ and +91-reverse 5’ATATAAGCTTCAACGGGAGTCACTCTCGAA-3’, and -758-forward 5’ATAT**CTCGAG**CTCCTCCTACCCGGATCA-3’ and +330-reverse ATATAAGCTTCGTTGCCCTGGTCGTTGGC were used, respectively. The XhoI and HindIII restriction enzyme sites were indicated in bold and underlined, respectively. The PCR products after purification were digested with XhoI/HindII enzymes to clone in the same site of the vector.

Site directed mutagenesis was carried out using the pGL4.79 based construct containing the -750 to +330 region of Hsp70A1A gene (isolated from dam methylase carrying *E*. *coli* strain) using the primer pair 5’ GCCCCAGCGAGTAGGTGGGGGC**GAA**CTCAATATCAAACTG-3’ (top strand) and 5’ CAGTTTGATATTGAGttcGCCCCCACCTACTCGCTGGGGC-3’ (botton strand) with the κB motif underlined. The mutated bases in the primer are written in the lower case. The amplification reaction with the primers was carried out in 50 μl reaction mix containing 100 ng template with 0.25 mM dNTP, 1 U pfu polymerase (Agilent,California, USA) under an optimised reaction condition (denaturation at 95°C/5 min once followed by 95°C/30 sec, annealing at 64°C/1 min and extension at 72°C/10 min) for 20 cycles. The PCR product after digestion with DpnI was used to transform *E*. *coli* and selected on ampicillin. The plasmid isolated from an isolated colony was confirmed to carry the mutation by nucleotide sequencing (Applied Biosystem).

### Construction of cell line stably expressing FLAG-Ku70 protein and transfection with the Hsp70A1A promoter deletion constructs

HeLa cells transfected using Lipofectamine 2000 (Life Tech, Invitrogen) with FLAG- Ku70 expression construct was subjected to the G418 selection starting at 24 h post transfection. Cells were maintained on G418 (700 μg/ml) for 21 days for the clonal selection of cells stably expressing FLAG-Ku70 protein as tested by immunoblot analysis using anti-FLAG antibody. Clones of cells expressing FLAG-Ku70 protein were amplified and stocked at liquid nitrogen. FLAG-Ku70 protein expression level was quantified with a known amount of FLAG-BAP (bacterial alkaline phosphatase) purchased from Sigma.

### Preparation of whole cell lysate and immunoblot analysis

Cells after treatments with heat shock (at 42°C/30 min) or not were allowed to recover for 45 min prior to harvesting on ice in ice cold PBS by scrapping with a policeman. After rinsing with ice-cold PBS to remove the growth media cells were lysed (in lysis buffer: 20 mM Tris-HCl pH 7.5, 1% Triton X-100, 150 mM NaCl, 5% glycerol, 1 mM phenylmethylsulfonyl fluoride (PMSF), 10 μg/mL leupeptin, 10 μg/mL aprotinin, 20 mM each of sodium fluoride and sodium vanadate). The lysate was centrifuged at 12,000*g* at 4°C for 15 min to collect the clear supernatant as the whole cell lysate (WCL). Total protein concentration in WCL was measured using the Bradford assay reagent (BioRad) with known amount of BSA as a standard. For a western blot, 50 μg or indicated amount of WCL was subjected to 5 or 10% sodium dodecyl sulfate polyacrylamide gel electrophoresis (SDS-PAGE) to transfer electrophoretically to polyvinylidene fluoride (PVDF) membranes (Millipore). After blocking in PBST (blocking buffer: PBS with 5% fat-free milk and 0.1% tween 20) for 20-min at room temperature, the membrane was incubated with primary antibody usually at 1:1000 or as appropriate dilution in the fresh blocking buffer for overnight at 4°C. The membrane was then washed 3–4 times with 30 ml for each wash with wash buffer (PBST) followed by incubation with a suitable secondary antibody (dilution 1:5000) 1 h at room temperature. The membrane was then washed as before and developed by chemiluminescent detection reagent by the manufacturer's instructions using Chemidoc (Biorad).

### Co-immunoprecipitation experiments

The whole cell lysate (usually 500 μg of protein per sample) was treated for 1 h with protein A/G agarose (10 μl bead volume) to remove the background (nonspecific interacting proteins). The clear supernatant collected was subjected to incubation with desired primary antibody anti-FLAG-M2 beads or anti-FLAG antibody at 4°C for 4 h with rolling condition followed by incubation (as appropriate) with 10 μl of protein A/G agarose for ~4 h. The pellet collected was rinsed a couple of times with lysis buffer, boiled in 1x Laemli buffer and processed for immunoblot with a desired antibody.

### Isolation of cytoplasmic and nuclear fractionation of cells

The (HeLa) cells were transfected with FLAG-p65 and his_6_-HSF1 expression constructs. Forty eight hours post transfection, cells were harvested in 1x PBS by scrapping with a policemen after treating with heat shock 42^°^C/30 min, or not. Cells subjected to heat shock were allowed to recover for 45 min at 37^°^C prior to harvesting. The cells were collected as pellet by centrifugation at 1000xg for 1 min at 4^°^C. The cytoplasm and nuclear fractions were isolated using NE-PER nuclear and cytoplasmic extraction kits as per manufacturer’s instruction (Thermo Scientific, USA).

### Immunofluorescence experiments

HeLa cells grown in 6 cm plates to ∼80% confluency were transfected with the FLAG-p65 and his_6_-HSF1 constructs. The cells were trypsinized 24 h post transfection to grow on the coverslip. Next day after subjecting to heat shock at 42^°^C/30 min (HS) or not (-HS) with or without 45 min recovery, coverslips were washed twice with PBS, fixed through treatment with 3.7% formaldehyde solution for 20 min. Following washing off the fixing solution with PBS, 0.2% titron X-100 was applied for 20 min (to enhance membrane permeabilization of cells). After blocking with 3% BSA, coverslips were incubated with anti-FLAG (1:250) or anti-his_6_ (1:250) antibody, washed, incubated with secondary antibody anti-mouse Alexa Flour 595 (1:100) or anti-rabbit TRITC (1:100), or anti-mouse Cyn5 (1:500) followed by incubation with DAPI (2 μg/ml) in the dark. After washing off the unbound materials with PBS, coverslips were mounted in DPX viewed and documented using a laser scanning confocal microscope (Leica TCS SP8, Leica Microsystems India Pvt. Ltd) using Leica Application Suite LAS X software.

### Densitometric and statistical Analysis

The densitometric analyses of the bands visualised by ethidium bromide staining in semi-quantitative RT-PCR experiments were performed by the ImageJ (NIH) and ImageLab (BioRad) softwares. Statistical significance was analysed by one way anova with Turkey’s post test or Student’s *t* test by Graphpad Prism 5.0 software. A p value of less than 0.05 was considered statistically significant.

## Results

### Downregulation of DNA-PK catalytic subunit affects the transcription of Hsp70A1A gene induced by heat shock treatment

Several evidence were considered prior to testing for the involvement of DNA-PK in the induction of Hsp70A1A transcription by thermal shock: i) we have indications that DNA-PK is retained with pre-initiation complex (PIC) assembled with the purified RNA polymerase II (pol II) and initiation factors on adenomajor late promoter when supplemented with HeLa nuclear extracts (M. Pal; unpublished data), ii) DNA-PK was shown to be involved in the expression of the estrogen and insulin responsive genes where it phosphorylates the respective activator protein [24,26,34], iii) DNA-PK mediated phosphorylation of TRIM28, a DNA damage associated protein facilitates pol II pause release on Hsp70A1B gene [25], and iv) demonstration of physical interaction with and phosphorylation of heat shock factor 1 by DNA-PK in the purified system [35].

**Fig 1:**
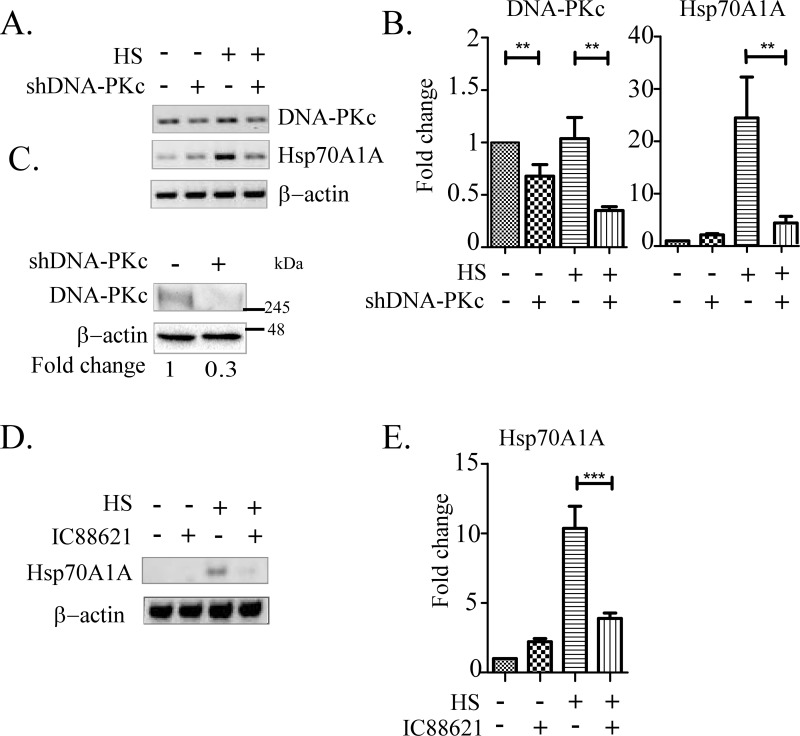
DNA-PKc is required for heat shock mediated transcription induction of Hsp70A1A gene in human cells. cDNAs prepared from HT1080 cells pretreated with shRNA against catalytic subunit of DNA-PK (shDNA-PKc) were subjected to PCR for testing its effects on the transcription of Hsp70A1A gene using the procedures described in the materials and methods. A) Representative ethidium bromide stained agarose gels showing relative transcript levels of DNA-PKc, Hsp70A1A and β-actin (as loading control). B) Estimation of DNA-PKc and the Hsp70A1A mRNA levels in indicated samples (indicated in panel A) by the RTq-PCR normalized with β-actin level. One-way ANOVA with Turkey’s post-test was used to analyse the data where **p < 0.01. C) Immunoblot showing downregulation of DNA-PKc protein in whole cell lysate isolated from HeLa cells pre-treated with shDNA-PKc or mock treated. Fold change shown was estimated by densitometric scanning of intensities of bands of DNA-PKc vs β-actin. D) Representative agarose gels stained with ethidium bromide showing the effect on Hsp70A1A transcription upon chemical inhibition (100 μM for 24 h) of DNA-PK determined by RT-PCR. E) Estimation of the effect of chemical inhibition DNA-PK by RT-qPCR assay on transcription of Hsp70A1A normalized with β-actin level. One-way ANOVA with Turkey’s post-test was used to analyze the data where **p < 0.001.

To test the role of DNA-PK in heat shock response (HSR), a shRNA-mediated downregulation of the catalytic subunit of DNA-PK (DNA-PKc) was carried out in HT1080 cells that carried a stably integrated tetracycline inducible shDNA-PKc cassette made in pTRIPZ vector through lentivirus mediated transduction. Treatment of these cells with doxycycline reduced DNA-PKc transcript level to ~60% of the normal level (not treated with Dox, Fig 1A and 1B). The effect on Hsp70A1A under this condition was estimated by treating these cells with or without heat shock by RT-PCR as well as RTq-PCR. As observed, treatment of cells under DNA-PKc downregulated condition with heat shock significantly reduced the transcription of Hsp70A1A chaperone gene (Fig 1A–1C). The involvement of DNA-PKc in Hsp70A1A transcription under heat shock was again confirmed by use of IC86621, a chemical inhibitor of DNA-PKc. It was found that pre-treatment of cells with IC86621 for 24 h reduced the heat shock mediated induction of Hsp70A1A by more than 2-fold (Fig 1D and 1E).

### Downregulation of Ku70 inhibits the transcription of Hsp70A1A gene upon heat shock treatment

**Fig 2:**
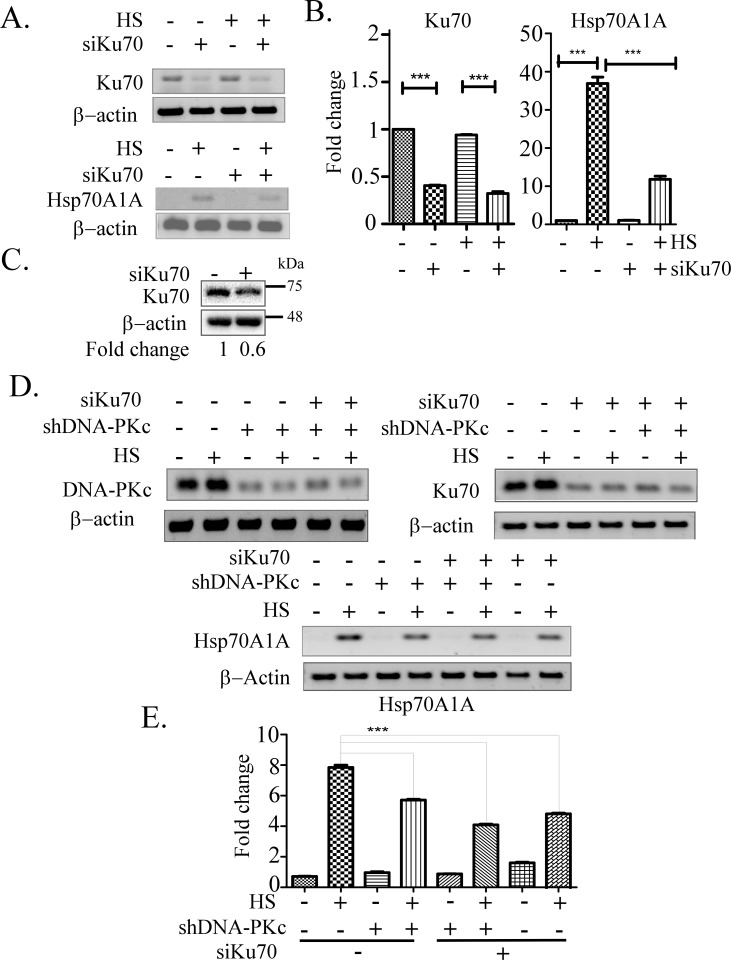
DNA-PK holoenzyme is required for induction of Hsp70A1A transcription in response to heat shock (HS). cDNAs prepared from cells pre-treated with Ku70 specific (siKu70), or scrambled siRNA were subjected to PCR to determine expression levels of indicated genes. A) Representative ethidium bromide stained agarose gels indicating the relative levels of Ku70 and Hsp70A1A transcripts. B) The abundance of the indicated transcripts/cDNAs (as indicated in panel A) were estimated by RT-qPCR normalized with normalized with β-actin level. One-way ANOVA with Turkey’s post-test was used to analyse the data where ***p < 0.001. C) Immunoblot showing downregulation of Ku70 protein in whole cell lysate isolated from HeLa cells pre-treated with siKu70 or scramble siRNA. Fold change shown was estimated by densitometric scanning of intensities of bands of Ku70 vs β-actin. D) Representative ethidium bromide stained agarose gels showing the levels of transcripts isolated from cells pre-treated with siKu70, or shDNA-PKc or both siKu70 and shDNA-PKc simultaneously determined by RT-PCR assay. E) Estimation of the intensities of bands shown in panel D through densitometric scanning. The β-actin level was used as the loading control (bottom panel). One-way ANOVA with Turkey’s post-test was used to analyse the data where ***p < 0.001.

The DNA-PK holoenzyme consists of DNA-PKc (catalytic subunit) and the regulatory Ku proteins (Ku70 and Ku86). The Ku70 and -86 subunits recruit the holoenzyme to the free double stranded DNA ends during double strand break repair (DBR) [36]. If DNA-PK holoenzyme is involved it is expected that Ku70 knock down would induce a similar effect on the expression of Hsp70A1A gene. It is also possible that the DNA-PKc and the Ku70 protein independently involved in the process [37]. The effect of downregulation of Ku70 transcript mediated by treatment with specific siRNA (siKu70) on expression of Hsp70A1A transcripts under heat shock was tested by semi-quantitative RT-PCR as well as RT-qPCR assay. We found that downregulation of Ku70 by more than 50% had compromised heat shock induced transcription of Hsp70A1A by more than 2-fold compared to mock treated samples based on an RT-PCR assay (Fig 2A–2C). Previously we tested that knockdown of DNA-PKc reduced Hsp70A1A transcription in response to heat shock by similar level (Fig 1B). Therefore, downregulation of both DNA-PKc and Ku70 affects induction of Hsp70A1A by a comparable level. Ku70 can have a DNA-PKc independent function in transcription. Or, Ku70 can also be involved here as a component of DNA-PK holoenzyme. To address that the cells were subjected to Ku70 and DNA-PK downregulation separately (Fig 2D top two panels) or simultaneously (Fig 2D, lower panel) and tested the resulting effect on the Hsp70A1A transcription under those condition by RT-PCR (Fig 2E). Because the effect of simultaneous knockdown of Ku70 and DNA-PKc were not significantly different from the effects by individual knockdown of those genes, the results support the conclusion that DNA-PK holoenzyme is involved in the transcription of Hsp70A1A under heat shock (Fig 2E).

### Heat shock dependent association of DNA-PK, and PARP-1 with p65/RelA

**Fig 3:**
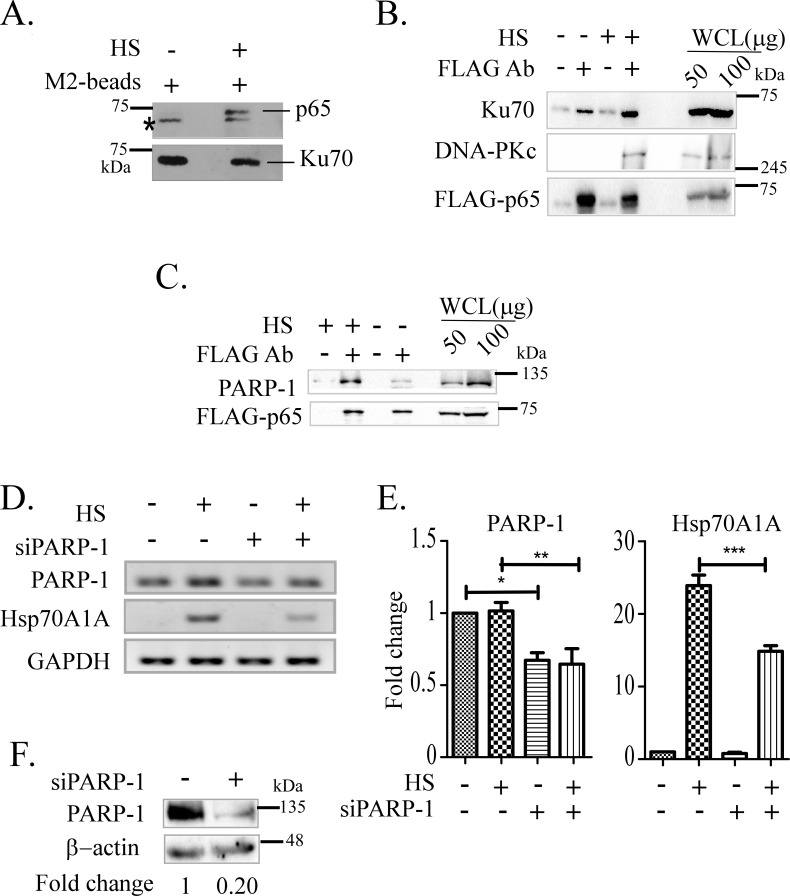
Interaction of p65/RelA with Ku70, DNA-PKc and PARP-1 determined by immunoprecipitation coupled immunoblot experiments. Whole cell lysates (WCL) of HeLa cells stably expressing FLAG-Ku70 or transiently expressing FLAG-p65/RelA proteins pretreated with heat shock (HS) or not were used. A) Detection of p65/RelA using anti-p65 antibody by immunoblot in immunoprecipitate (IP) obtained from WCL of cells stably expressing FLAG-ku70 using anti-FLAG (M2) antibody. Asterisk denotes a non-specific band. The same membrane was stripped to detect the presence of FLAG-Ku70 protein (lower panel) using anti-FLAG antibody. B) Immunoblot to detect Ku70 or DNA-PKc in immunoprecipitate isolated with anti-FLAG antibody from WCL of cells expressing FLAG-p65/RelA pretreated as indicated. FLAG-p65/RelA level was detected by reblotting the same membrane with the anti-FLAG antibody (FLAG-Ab). The amounts of WCL analyzed as an internal control to identify the target protein band were indicated on the right. C) Immunoblot showing PARP-1 in immunoprecipitate isolated from WCL used in panel B. D) RT-PCR analysis of cDNAs prepared from cells treated with PARP-1 specific siRNA (siPARP-1) or mock to show the effects on Hsp70A1A transcription as indicated by representative agarose gels stained with ethidium bromide. E) The abundance of the indicated transcripts/cDNAs (indicated in panel D) were estimated by RT-qPCR normalized with GAPDH level as internal control. One-way ANOVA with Turkey’s post-test was used to analyse the data where *p < 0.05, **p < 0.01 and ***p < 0.001 respectively. F) Immunoblot showing downregulation of PARP-1 protein in WCL isolated from HeLa cells pre-treated with si-PARP-1 or mock treated. Fold change shown was estimated by densitometric scanning of PARP-1 vs β-Actin bands.

Having found that knockdown of both DNA-PKc and Ku70 affects Hsp70A1A expression under heat shock, it was logical to test if DNA-PK holoenzyme, i.e., the DNA-PKc and Ku70 interacts with any of the components implicated in the heat shock response pathway such as HSF1 [35]. Earlier DNA-PK was shown to be required for activities of estrogen receptor and USF-1 in estrogen and insulin signalling pathways, respectively [24,34]. Immunoprecipitation experiments were carried out to detect Ku70 associated protein under thermal shock. HeLa cells stably expressing FLAG-epitope tagged Ku70 (FLAG-Ku70) were subjected to heat shock for 30 min or not followed by 45 min recovery, and FLAG-Ku70 associated proteins in the whole cell lysates (WCL) were immunoprecipitated using anti-FLAG antibody. The immunoprecipitates after washing off the unbound materials were resolved in SDS-PAGE and immunoblotted using antibodies against different transcription factors such as HSF1, HIF1α, p53, and p65/RelA. Immunoblotting with HSF1 along with HIF1α, or p53 antibody did not yield any positive result. However, as shown in Fig 3A, the presence of the p65/RelA was exclusively detected in immunoprecipitate isolated from WCL of cells pre-treated with heat shock compared to the control. The presence of Ku70 level in those samples was tested by reblotting the membrane with anti-FLAG antibody. The results indicated that Ku70 forms a complex with p65/RelA under heat shock treatment (Fig 3A, bottom panel). The presence of p65/RelA in the immunoprecipitate of WCL isolated from two additional cell lines HT1080 and HCT116 pretreated with heat shock was detected by immunoblot experiments (data not shown). These results indicated that this interaction is not specific to a particular cell line. Association of p65/RelA with Ku70 was confirmed by determining the presence of Ku70 by immunoblot in p65/RelA associated immunoprecipitate (Fig 3B). The presence of DNA-PKc in the same immunoprecipitate was also detected by immunoblot with the anti-DNA-PKc antibody (Fig 3B). These results, therefore, are consistent with the idea that DNA-PK holoenzyme forms complex with the p65/RelA upon treating the cells with heat shock.

Both in DNA repair, as well as transcription regulation PARP-1 was involved with DNA-PK [32]. To understand if PARP-1 occurs in the same complex immunoprecipitation experiment was carried out with WCL isolated from cells transiently transfected with FLAG-p65 expression construct pretreated with heat shock and no heat shock. As expected, immunoblot experiment with anti-PARP-1 antibody detected the relatively increased presence of PARP-1 with FLAG-p65 following heat shock treatment indicating the association of PARP-1 with p65/RelA complex along with DNA-PK holoenzyme (Fig 3C). To test its requirement in heat shock induced transcription of Hsp70A1A cells were treated with PARP-1 siRNA or scramble siRNA followed by treatment with heat shock. Downregulation of PARP-1 in transcript and protein level was estimated by qRT-PCR as well as immunoblot experiments prior to assess the effect on Hsp70A1A transcription (Fig 3D–3F). As shown, downregulation of PARP-1 reduced Hsp70A1A gene transcription under heat shock by 50% where the knockdown of PARP-1 mRNA and protein was achieved to be about 30% and 80%, respectively (Fig 3D–3F). Dependence of Hsp70A1A transcription on DNA-PK and PARP-1 was indicative of an involvement of double stranded DNA break on the gene. In fact, earlier studies with estrogen, insulin, and androgen induced gene regulation showed involvement TopoIIβ, along with DNA-PK and PARP-1. As such the enzymatic function of TopoIIβ is responsible for releasing topological barrier on DNA through creating transient double stranded break [25,32]. The requirement of TopoIIβ was tested by inhibiting its enzymatic function by two chemicals, merbarone, and etoposide which block its function through blocking DNA binding and ligation function, respectively [38]. As shown by RT-PCR based assay that treatment of cells with both of these chemical inhibitors reduced the inducibility of Hsp70A1A transcription by heat shock treatment (S1 Fig). These results are consistent with the idea that upregulation of Hsp70A1A also is dependent on TopoIIβ along with DNA-PK and PARP-1.

### NFκB (p65/RelA-p50) is required for transcription of Hsp70A1A gene induced by heat shock treatment

**Fig 4:**
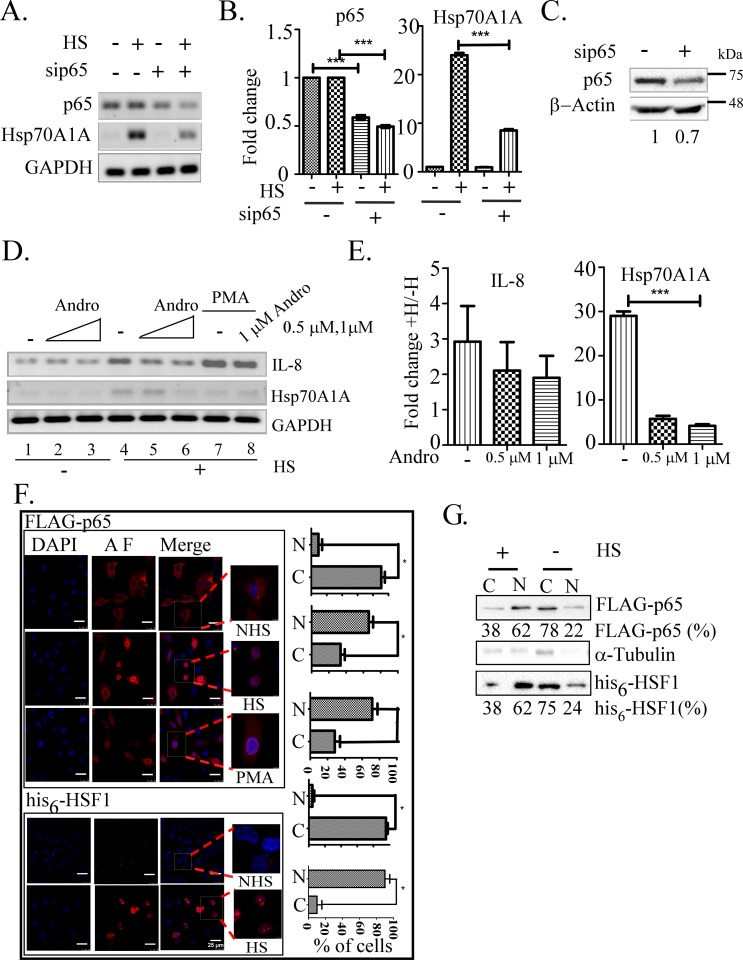
The requirement of p65/RelA in Hsp70A1A transcription under heat shock. A) Representative ethidium bromide stained agarose gels showing relative transcripts levels prepared from HeLa cells as indicated determined by RT-PCR. B) Estimation of the effect of p65 knockdown on Hsp70A1A transcription shown in panel A by RT-qPCR using GAPDH as an internal control. One-way ANOVA with Turkey’s post test was used to analyse the data where ***p < 0.001. C) Immunoblot showing downregulation of p65/RelA protein in WCL isolated from HeLa cells pre-treated with sip65 or scramble siRNA. Fold change shown was estimated by densitometric scanning of intensities of bands of p65/RelA vs β-actin. D) Representative ethidium bromide stained agarose gels showing the effects on Hsp70A1A following the treatment of cells as indicated. PMA treated cells were included as positive control. Andro, andrographolide. E) Estimation of intensities of the bands in panel D. GAPDH level was measured as an internal control. One-way ANOVA with Turkey’s post test was used to analyse the data where ***p < 0.001. F) p65/RelA presence in the nucleus following heat shock (plus 45 min recovery at 37°C) or PMA treatment detected by indirect immunofluorescence technique visualised by a confocal microscope. Antibodies against the epitopes were used to detect transiently expressed the FLAG-p65 or his_6_-HSF1 protein, respectively. The images were taken in 63x magnification with a 3x optical zoom. HS, heat shock; NHS, non heat shock. AF, alexa flour. The bar graph on the right show the estimates of cells (%) with nuclear p65. Estimation was carried out using student’s *t* test, *p<0.05. G) Translocation of FLAG-p65 and his_6_-HSF1 in isolated cytoplasmic (C) and nuclear (N) protein fractions by immunoblot using anti-FLAG and anti-his_6_ antibody, respectively. The α-tubulin protein level was estimated by immunoblot as a cytoplasmic protein marker. Net nuclear translocation of p65/RelA and his_6_HSF1 was estimated by subtracting residual cytoplasm contamination in the nuclear fraction equivalent to α-tubulin level.

To test if p65/RelA is important in heat shock induced transcription of Hsp70A1A gene, cells were treated with siRNA against p65/RelA followed by treating the cell by heat shock or not. Both semi- as well as quantitative RT-PCR, was carried out to test the effect on the Hsp70A1A expression. That Hsp70A1A level was compromised by more than 2-fold under a condition when p65/RelA knockdown was effective by about 30% supporting the involvement of p65/RelA in Hsp70A1A transcription under heat shock treatment (Fig 4A–4C). To understand if here p65-p50 form is involved, the expression of Hsp70A1A gene was tested upon heat shock in cells pretreated with andrographolide, an inhibitor of p50/NFκB1 [39]. To this end cells pre-treated with different concentrations of andrographolide (Andro) for overnight were subjected to heat shock treatment or not. As a positive control for andrographolide function, cells pre-treated with andrographolide were also treated with PMA as an inducer of NFκB function. We found that treatment with andrographolide compromised expression of Hsp70A1A in a dose-dependent manner (Fig 4D, compare lanes 1–3 with 4–6). The NFκB inhibitory function of andrographolide was confirmed by inhibition of IL8 transcription induced by PMA treatment (Fig 4D, lanes 7–8). The effect was estimated by RT-qPCR (Fig 4E). Therefore, a p65-p50 form of NFκB may be involved in regulation of Hsp70A1A expression under heat shock.

NFκB resides in the cytoplasm under normal condition. We tested if NFκB translocates to the nucleus with heat shock (Fig 4F). Distribution of FLAG-p65/RelA and his_6_-HSF1 as positive control was monitored in cells transiently transfected simultaneously with both expression constructs after 45 min recovery following heat shock for 30 min. Cells were treated with PMA to test p65/RelA translocation as a positive control as well. Nuclear-cytoplasmic distribution of these proteins was monitored by confocal microscopy after immunostaining with fluorescence labeled epitope-specific antibodies. As shown FLAG-p65/RelA was translocated to the nucleus with heat shock followed by recovery treatment alike translocation of his_6_-HSF1, or p65/RelA treated with PMA (Fig 4F). Weak or no his_6_-HSF1 signal was visible in non heat shock treated sample may be indicative of the unavailability of his_6_ epitope to the anti-his_6_ antibody used. This result is consistent with the idea that his_6_-epitope is exposed after heat shock when HSF1 forms homotrimer [40]. NFκB/p65 translocation to nuclei upon heat shock under similar condition was estimated to be alike HSF1 by immunoblot experiment (Fig 4G). Therefore, the involvement of NFκB in the Hsp70A1A gene transcription was supported by its translocation to the nucleus after heat shock treatment.

### Evidence that NFκB controls Hsp70A1A promoter under heat shock

**Fig 5:**
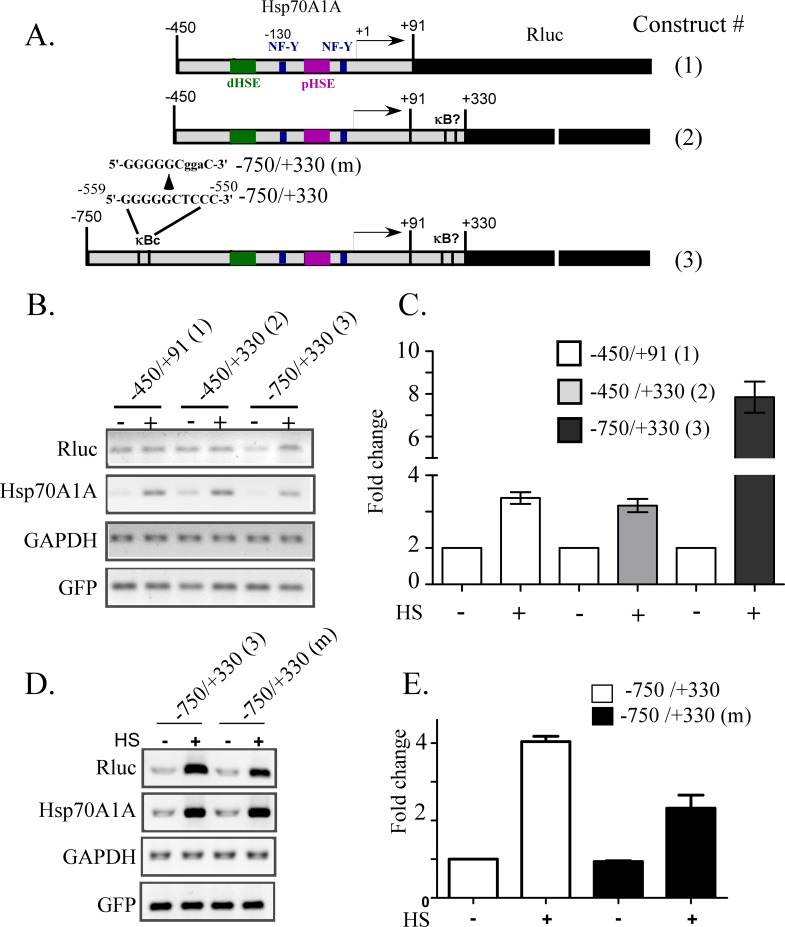
NFκB modulates Hsp70A1A promoter activity under heat shock. A) Schematic showing the regions of Hsp70A1A promoter fragments cloned upstream of renila luciferase reporter (Rluc). The locations of heat shock elements (dHSE, distal heat shock element; pHSE, proximal heat shock element), NF-Y and κB consensus sequence (κBc) and a less conserved κB (?) site are indicated with respect to transcription start site (+1, an arrow). The direction of the arrow indicates the direction of transcription. B) RT-PCR assay with transcripts prepared from HEK293 cells 48 h post transfection with the constructs shown in panel ‘A’. Representative ethidium bromide stained agarose gels showing relative levels of indicated transcripts. C) Estimation of the band intensities of luciferase cDNA vs those of GFP and GAPDH as the internal control. D) RT-PCR assay with transcripts prepared from HEK293 cells 48 h posttransfection with the constructs indicated (also shown in panel A, constructs #3 and #3(M)). Representative ethidium bromide stained agarose gels showing relative levels of transcripts as indicated. E) Estimation of the band intensities of lucifearse cDNA vs those of GFP and GAPDH as the internal control. HS, heat shock.

Association of p65/RelA with DNA-PK and PARP-1, and requirement of p65/RelA for Hsp70A1A transcription under heat shock is suggestive of the role of NFκB in the Hsp70A1A promoter regulation (Figs 3 and 4). The nucleotide sequence of Hsp70A1A promoter was scanned for a probable NFκB recognition κB motif. Analyses of the nucleotide sequence using bioinformatics tools as well as manual inspection identified a consensus κB motif at—559/-550 with a relatively less probable site at location +294/+303, where +1 is the transcription start site. To determine if any of these two elements are functional under heat shock reporter constructs were made by inserting three different partially deleted fragments of Hsp70A1A promoter DNA upstream of the renila luciferase reporter (Fig 5A). Cells transfected with these reporter plasmids along with pCMV-GFP reporter (pLVGFP) construct [41] as internal transfection control were treated with heat shock or not to evaluate by RT-qPCR the levels of luciferase transcript (no recovery was given after heat shock). Cells carrying these reporter plasmids were exposed to similar treatment (48 h post transfection) is justifiable by similar response of their endogenous Hsp70A1A gene. Similar levels of GAPDH and GFP transcript in all samples also validated the high confidence in the data (Fig 5B and 5C). The presence in the promoter the segment between -750/-450 that carried only one consensus κB at -559/-550 induced the reporter by about eight-fold compared to only two-fold induction by heat shock in its absence (Fig 5B and 5C). The reduction of promoter activity can be attributed to the absence of κB element because no other recognition motifs for a transcription factor such as CREB, NF-Y and HSE were uniquely located in the region. The sequence motifs for these factors identified on the Hsp70A1A promoter were indicated (Fig 5A). To test if the NFκB dependent stimulation of activity by heat shock is through the κB motif at -590/-550, a reporter construct was created (-750/+330(m)) where the nucleotide sequence of κB motif 5’(-559)GGGGGCTCCC(-550)-3’ in the -750/+330 construct was changed to 5’(-559)GGGGGggaC(-550) by site directed mutagenesis (Fig 5A). As expected, cells transfected with this mutant construct compromised luciferase reporter transcription under heat shock by about two-fold (Fig 5D and 5E, compare -750/+330 with -750/+330(m)). Because the nucleotide sequence of this mutant promoter only differed in three nucleotides as indicated the reduction of luciferase transcription under heat shock is most likely due to loss of NFkB binding to the Hsp70A1A promoter. Attempts were made to estimate heat shock induced p65/RelA recruitment to this κB site by chromatin immunoprcipitation (ChIP) but were unsuccessful. This might be due to inefficient formaldehyde crosslinking or inaccessibility of the epitope of FLAG-p65 by anti-FLAG antibody used during immunoprecipiation, or both (data not shown).

Immunofluorescence experiment coupled with confocal microscopy was carried out to check the nuclear distribution of p65/RelA with HeLa cells transiently transfected with FLAG-p65/RelA expression construct (without recovery). As a positive control his_6_-HSF1 expression construct was mixed with FLAG-p65/RelA during transfection. As shown clear translocation of p65/RelA into the nucleus was visible in some of the transfected cells stained with Alexa Flour 595 (S2 Fig). About 50% transiently transfecetd cells expressing p65/RelA carried the protein in the nucleus. Response of the cell to heat shock treatment was confirmed by efficient translocation of HSF1 into nucleus in all transfected cells stained with TRITC (S2 Fig). Taken together these results suggest that the consensus κB site is utilised by NFκB upon heat shock and the interaction is important in Hsp70A1A transcription under heat shock stress.

## Discussion

Hsp70A1A, a member of molecular chaperones plays important role in different cellular processes including thermotolerance, clearance of protein aggregate, survival and proliferation. We report here an involvement of DNA-PK, PARP-1, TopoIIβ, and NFκB during Hsp70A1A transcription following heat shock. We also show that DNA-PK, PARP-1 associate with NFκB (p50-p65) under the same condition. Sequence analysis identified a consensus κB sequence motif on the Hsp70A1A promoter and deletion of this element compromised its induction by heat shock. The results are consistent with the idea that DNA-PK and PARP-1 associate with the Hsp70A1A promoter through complexation with NFκB assembled on the Hsp70A1A promoter under heat shock treatment. These studies reveal an involvement of NFκB with the DRB complex for the first time in Hsp70A1A transcription activation under thermal shock.

Role of DNA-PK, TopoIIβ and PARP-1 as a part of DNA-break repair complex as well as transcription activation complex was reported earlier [23,24,25]. Various modes of association of NFκB with DNA-PK and PARP-1 have been reported earlier; an interaction of DNA-PK with p50/NFκB was reported in VCAM-1 gene transcription in response to TNF treatment. It was shown that DNA-PK mediated phosphorylation of p50 increases DNA-binding affinity of p50-p65 as well as p50-p50 to VCAM-1 promoter [37]. Interaction of PARP-1 with NFκB during its inflammatory response has been shown previously as well [42,43]. PARP-1 was shown to interact physically with both p50 and p65/RelA subunits of NFκB [44]. It was shown to be a gene specific coactivator for NFκB [44]. Acetylation of PARP-1 by p300/CBP was shown to be required for NFκB function in response to a proinflammatory stimulation [32,45]. Interaction of DNA-PK and PARP-1 was suggested by the occurrence of PARylated DNA-PK that is essential for a specific function of DNA-PK [46]. Formation of a complex by direct association of PARP-1 with Ku70/80 was implicated in binding with enhanced affinity to unpaired DNA region in the genome [46]. These results sustain possibility of association of NFκB with DNA-PK and PARP-1 through multiple interactions. The assembled complex presumably brings the DNA-PK physically in close proximity to transcription complex or/and for transcription factor for modulation of activity. Interaction of components of DNA-PK holoenzyme with HSF1 trimer was shown to be stimulatory of DNA-PK activity [35,47]. DNA-PK was shown to phosphorylate RNA polymerase II and participate in receptor mediated transcription activation involving estrogen receptor and USF-1, respectively [24,34,48]. DNA-PK was also shown to be recruited at the coding region to facilitate RNA polymerase II pause release on Hsp70A1B gene [25]. We observed an induction of the phosphorylation of CTD at serine 2 of Rpb1 of RNA pol II by heat shock in DNA-PK dependent manner because it was inhibited by chemical inhibition of DNA-PK (S3 Fig). However, in the same sample the role of DNA-PK in the enhanced phosphorylation of HSF1 in response to heat shock was not detectable (S3 Fig). This may be due to the method used here, which only can detect relatively a large change in the MW or mobility of a protein due to multiple phosphorylation events on it. If DNA-PK only phosphorylates a single amino acid on HSF1 that can possibly be detected by two- dimensional gel electrophoresis.

Inducible gene expression was shown to accompany double stranded DNA break in the promoter as well as in the coding region [24,25,34]. The break was generated through participation of topoisomerase IIβ (topoIIβ) induced by topological constrains developed due to various activities on the DNA such as replication, chromosome segregation, compaction and transcription [23,32,49,50]. It is conceivable that generation of DNA free ends allows DNA-PKc approach physically close to the promoter or the transcription complex through its regulatory Ku subunits. Recently, the involvement of a similar situation on Hsp70A1B gene under heat shock, implicated in RNA polymerase II transcription pause release, was noticed. Here, the DNA break was mapped to the transcribing region of Hsp70A1B upon induction by different stressors including heat shock [25]. Given the similarity of response to heat shock treatment it is possible that DNA break is generated on allowing DBR complex to dock on the Hsp70A1A gene. Indeed, under the heat shock condition used in this study like Hsp70A1A, Hsp70A1B transcription was found to be similarly sensitive to the cellular downregulation of DNA-PKc and p65/RelA (S4 Fig). Heat shock condition used was also found to induce the celluar level of γ-H2A.X phosphorylation determined by immunoblot assay (S5 Fig). In addition, chemical inhibition of TopoIIβ reduced the induction of Hsp70A1A significantly (S1 Fig).

The most common form of NFκB is constituted by p65/RelA and p50 heterodimers which bind to nucleotide sequence κB motif of consensus sequence 5’-GGGRNYYYCC-3’ (where R is a purine, N is any nucleotide and Y is a pyrimidine) [51,52]. Scanning of nucleotide sequence of the Hsp70A1A gene identified a consensus NFκB recognition motif at -559/-550, (+1, the transcription start site). NFκB composed of p65/RelA and p50 was thought to be involved because inhibition of both p65/RelA and p50 affected the induction of Hsp70A1A transcription upon heat shock (Fig 4). The presence of a κB motif suggests an engagement mechanism for NFκB on the Hsp70A1A promoter. An association of NFκB with the DBR complex is suggestive of recruitment of DBR complex to the Hsp70A1A promoter under this condition. It remains to be tested if this interaction is facilitated by binding of NFκB to κB motif on the Hsp70A1A promoter DNA. Nevertheless, our immunoprecipitation results suggest that NFκB as part of the DBR can also localise itself on the DNA break site through Ku subunits of DNA-PK (Fig 3).

Modulation of NFκB activity by DNA-PK following DNA damage has been studied by several groups. It was shown that DNA-PK was required to activate NFκB following DNA damage induced by IR radiation which involve phosphorylation of IκBα [53]. Role of DNA-PK in NFκB activation in response to DNA break induced by treatment with a topoII inhibitor N-benzyl-adriamycin has been reported [54]. Panta et al showed that DNA-PK activated this way activates MAPK/p90^rsk^ signalling cascade and the activated p90^rsk^ in turn activate IκB kinase 2 to activate NFκB [54]. In contrary to these results Liu et al have found that DNA-PK mediated phosphorylation of IκBα could inhibit NFκB activation [55].

The involvement of NFκB in Hsp70A1A transcription is consistent with their cellular roles as both of these proteins support cellular survival and proliferation through supporting anti-apoptotic pathway [56,57]. NFκB has been shown to regulate both the human Hsp90α and mouse Hsp70 genes [58,59]. The mouse and human Hsp70A1A promoter and UTR are ~51% similar in sequence indicating that the occurrence of NFκB on the mouse has not made it obvious that the human gene will also be regulated by the NFκB. Indeed, cardioprotective role of NFκB through upregulation of Hsp70 during late phase ischemic heart preconditioning was shown in mice [5,60]. It is conceivable that cells have developed this strategy to link the pro-inflammatory factor with the molecular chaperones to build cooperative activities for the benefit of the cells in need. It remains to be seen if NFκB on the human Hsp70A1A is engaged in the coordination of activity with additional transcription factors as observed in the case of mouse Hsp70 gene [59]. Exposure to thermal shock produces toxic protein aggregates in cells. Indeed involvement of NFκB has been implicated in the recovery of the cells from heat induced damage through induction of protein quality control apparatus as well as anti-apoptotic signalling following thermal stress [57,61,62].

In summary, these studies revealed important role of DBR complex carrying DNA-PK, PARP-1 and TopoIIβ in heat shock response. Results obtained are consistent with the idea that NFκB while bound to its recognition sequence κB on the Hsp70A1A promoter can engage the DRB complex spatially in close proximity to its (DRB) targets under heat shock. Further study is required to understand how NFκB establishes the interaction(s) with the DBR complex and the nature of the targets involved.

## Supporting Information

S1 Fig Treatment of cells with TopoIIβ inhibitor merbarone and etoposide inhibits heat shock dependent activation of Hsp70A1A. Representative agarose gels stained with ethidium bromide showing relative transcript levels of Hsp70A1A gene in HeLa cells pretreated with vehicle (DMSO) of 50 μM merbarone (Mer) (A) or 20 μM etoposide (Eto) (B) for indicated period determined by RT-PCR. GAPDH levels were estimated as a loading control, C) Bar graph showing the estimation of band intensities in (A) through densitometric scanning.
(TIF)

S2 Fig Subcellular distribution of p65/RelA in HeLa cells immediately after heat shock determined by indirect immunofluorescence. HeLa cells carrying FLAG-p65 and his_6_-HSF1 expression constructs pretreated with heat shock (HS) or no heat shock (NHS) were stained with the indicated eptitope specific antibodies to show their subcellular distribution. Cells were treated with PMA to show the translocation of p65/RelA as a positive control. The images were taken in 63x magnifications with a 2x optical zoom.
(TIF)

S3 Fig Effect of DNA-PK on ser-2 phosphorylation of Rpb1 c-terminal domain and heat shock factor 1 (HSF1) induced by heat shock treatment. Immunoblots with indicated antibodies using the whole cell lysates prepared from his_6_-HSF1 expressing HeLa cells pretreated with DNA-PK inhibitor IC88621 (100 μM) for 24 h or the vehicle followed by heat shock (HS) treatment or not. Lanes (lanes 5–8) corresponding to CIP carried samples pretreated with calf intestinal phosphatase. The β-actin levels were determined as internal loading control.
(TIF)

S4 Fig Knockdown of DNA-PKc and p65/RelA affects Hsp70A1B and Hsp70A1A to similar extent. Representative ethidium bromide stained agarose gel indicating relative transcript levels in HeLa cells pre-treated with shDNA-PKc or sip65/ReLA following heat shock determined by RT-PCR assay. Panel (A) represents shDNA-PK pretreated samples. Panel (B) represents sip65/RelA pretreated samples. GAPDH levels were determined as an internal loading control.
(TIF)

S5 Fig Heat shock induces DNA break as revealed by elevated levels of γH2A.X phosphorylation under heat shock. Immunoblot showing levels of phosphorylated γH2A.X protein in whole cell lysates isolated from HeLa cells that were subjected to different duration (0, 15, 30 min) of heat shock treatment or not. β-actin level was determined as internal loading control.
(TIF)
