# Supplementary figures and images for: Engagement of Components of DNA-Break Repair Complex and NFκB in Hsp70A1A Transcription Upregulation by Heat Shock
Source: PLoS One. 2017 Jan 18;12(1):e0168165. doi: 10.1371/journal.pone.0168165 (PMC5242496; doi:10.1371/journal.pone.0168165)

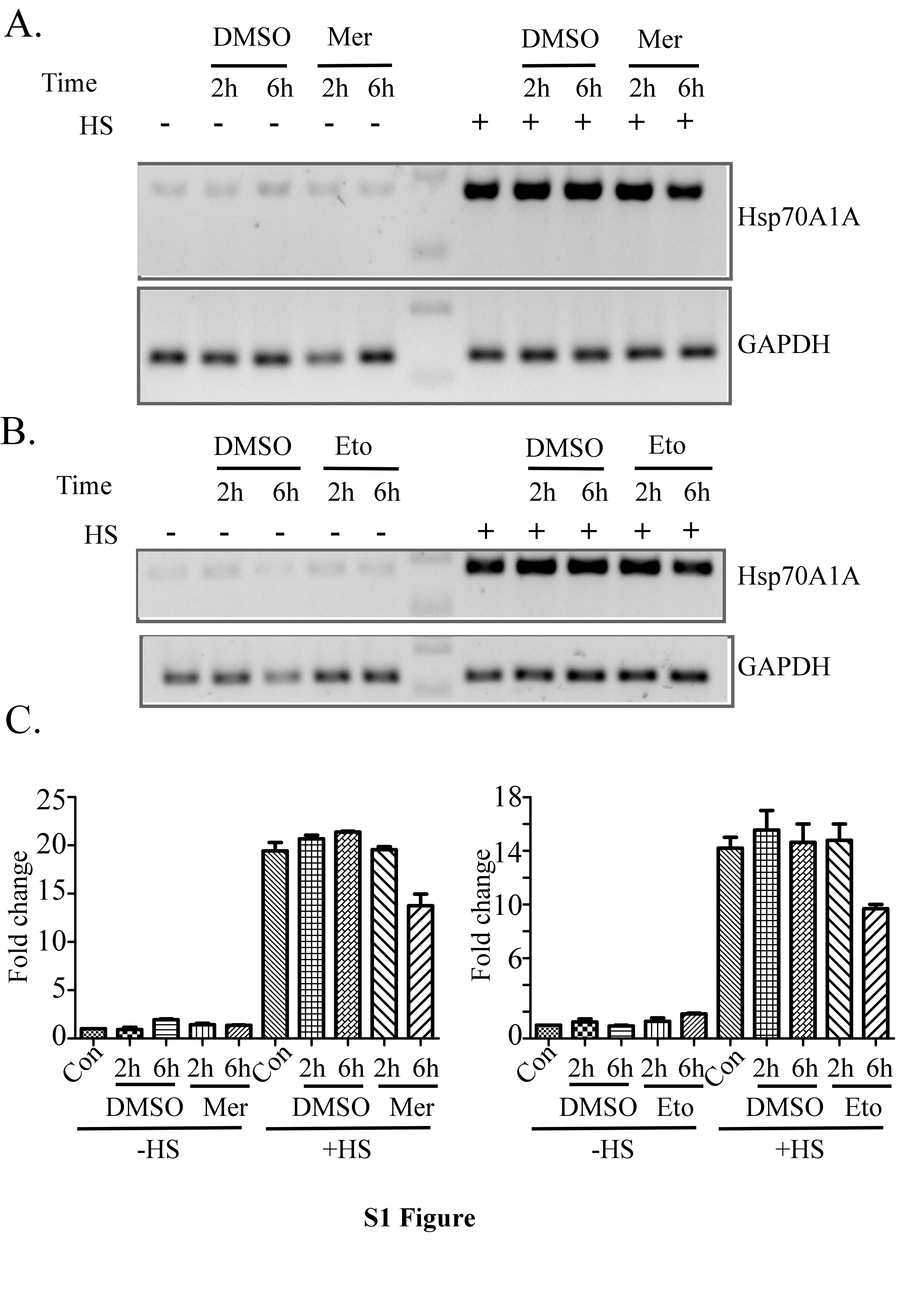

Supplement: S1 Fig — Representative agarose gels stained with ethidium bromide showing relative transcript levels of Hsp70A1A gene in HeLa cells pretreated with vehicle (DMSO) of 50 μM merbarone (Mer) (A) or 20 μM etoposide (Eto) (B) for indicated period determined by RT-PCR. GAPDH levels were estimated as a loading control, C) Bar graph showing the estimation of band intensities in (A) through densitometric scanning. (TIF) [file pone.0168165.s001.tif]

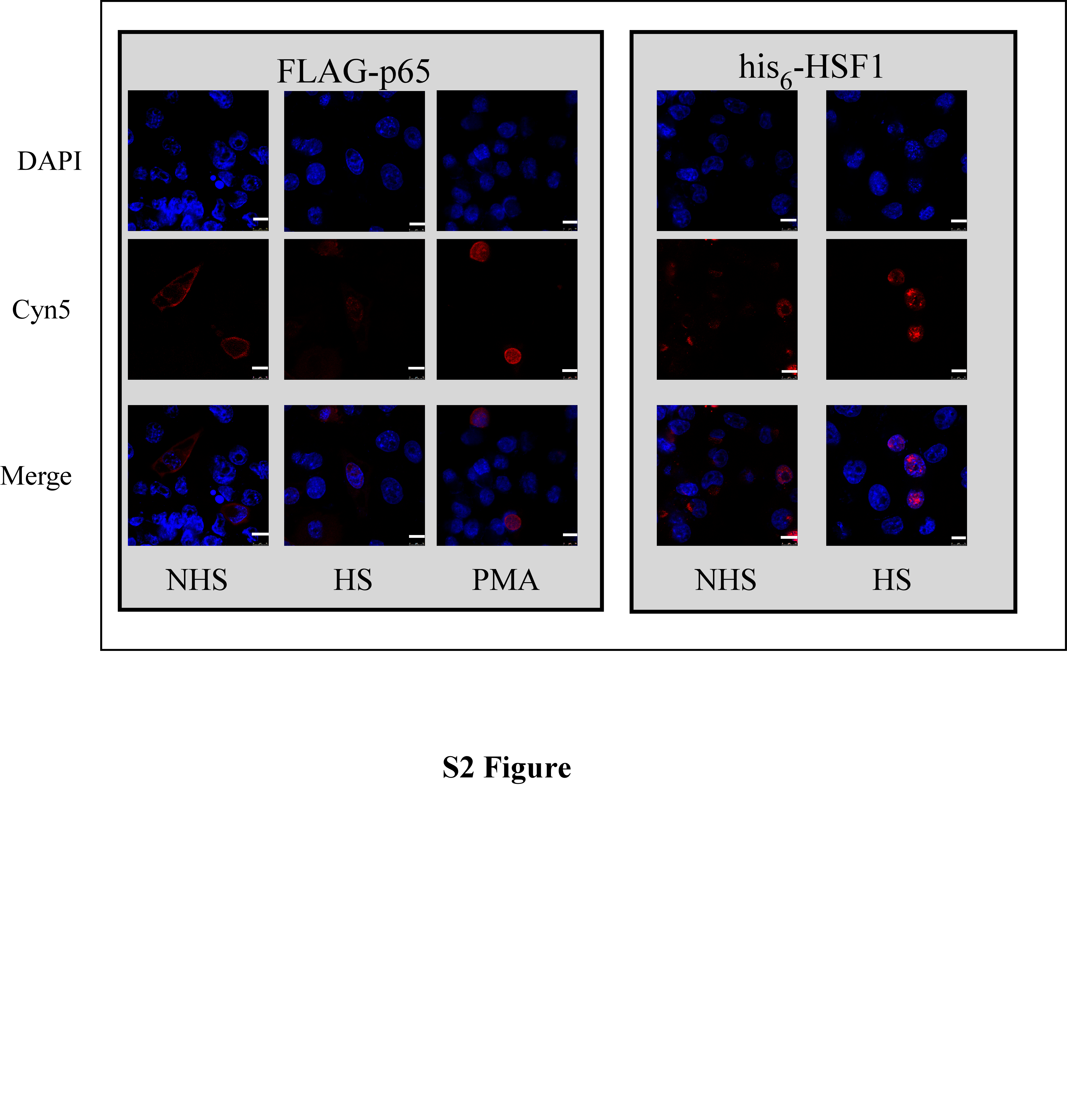

Supplement: S2 Fig — HeLa cells carrying FLAG-p65 and his6-HSF1 expression constructs pretreated with heat shock (HS) or no heat shock (NHS) were stained with the indicated eptitope specific antibodies to show their subcellular distribution. Cells were treated with PMA to show the translocation of p65/RelA as a positive control. The images were taken in 63x magnifications with a 2x optical zoom. (TIF) [file pone.0168165.s002.tif]

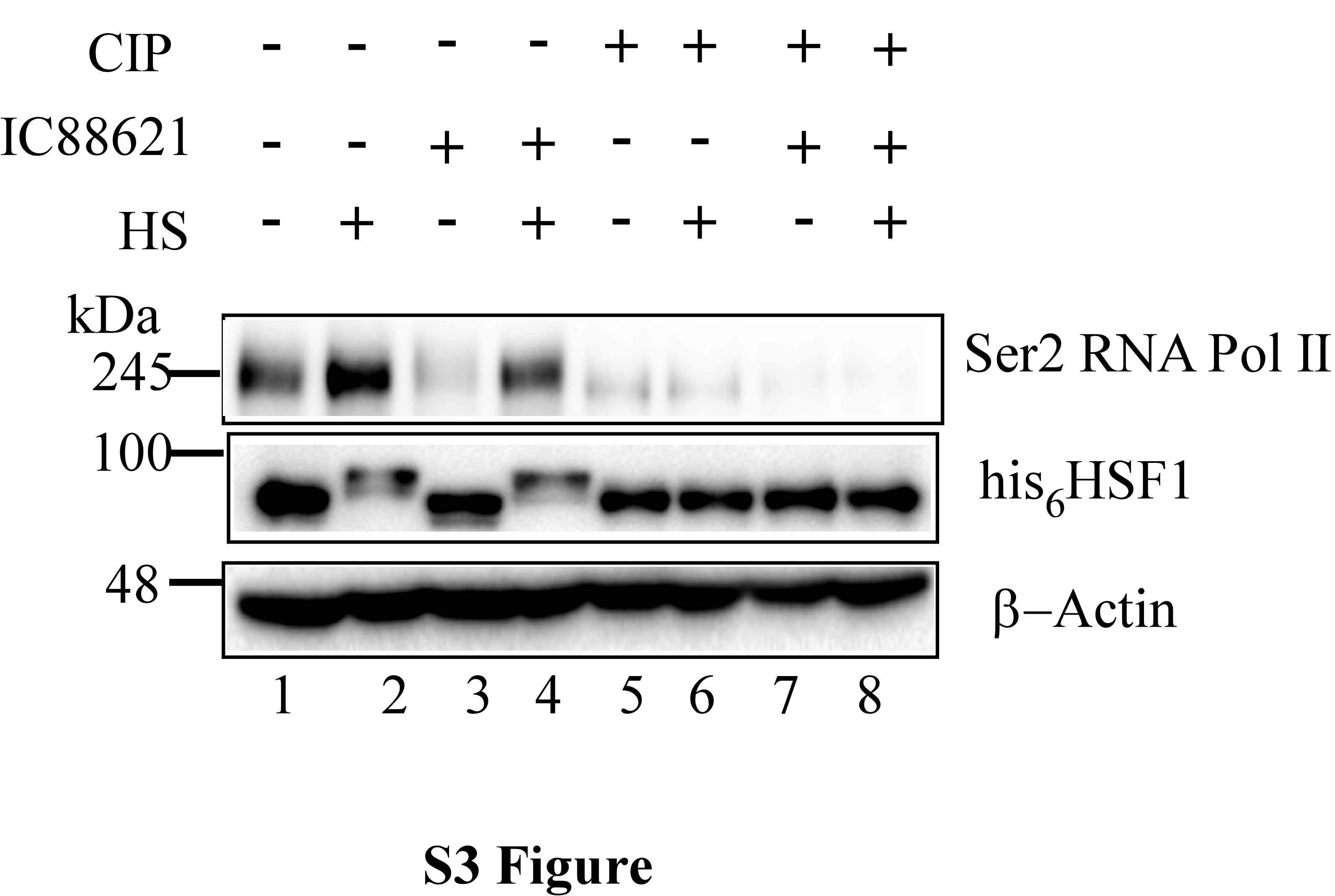

Supplement: S3 Fig — Immunoblots with indicated antibodies using the whole cell lysates prepared from his6-HSF1 expressing HeLa cells pretreated with DNA-PK inhibitor IC88621 (100 μM) for 24 h or the vehicle followed by heat shock (HS) treatment or not. Lanes (lanes 5–8) corresponding to CIP carried samples pretreated with calf intestinal phosphatase. The β-actin levels were determined as internal loading control. (TIF) [file pone.0168165.s003.tif]

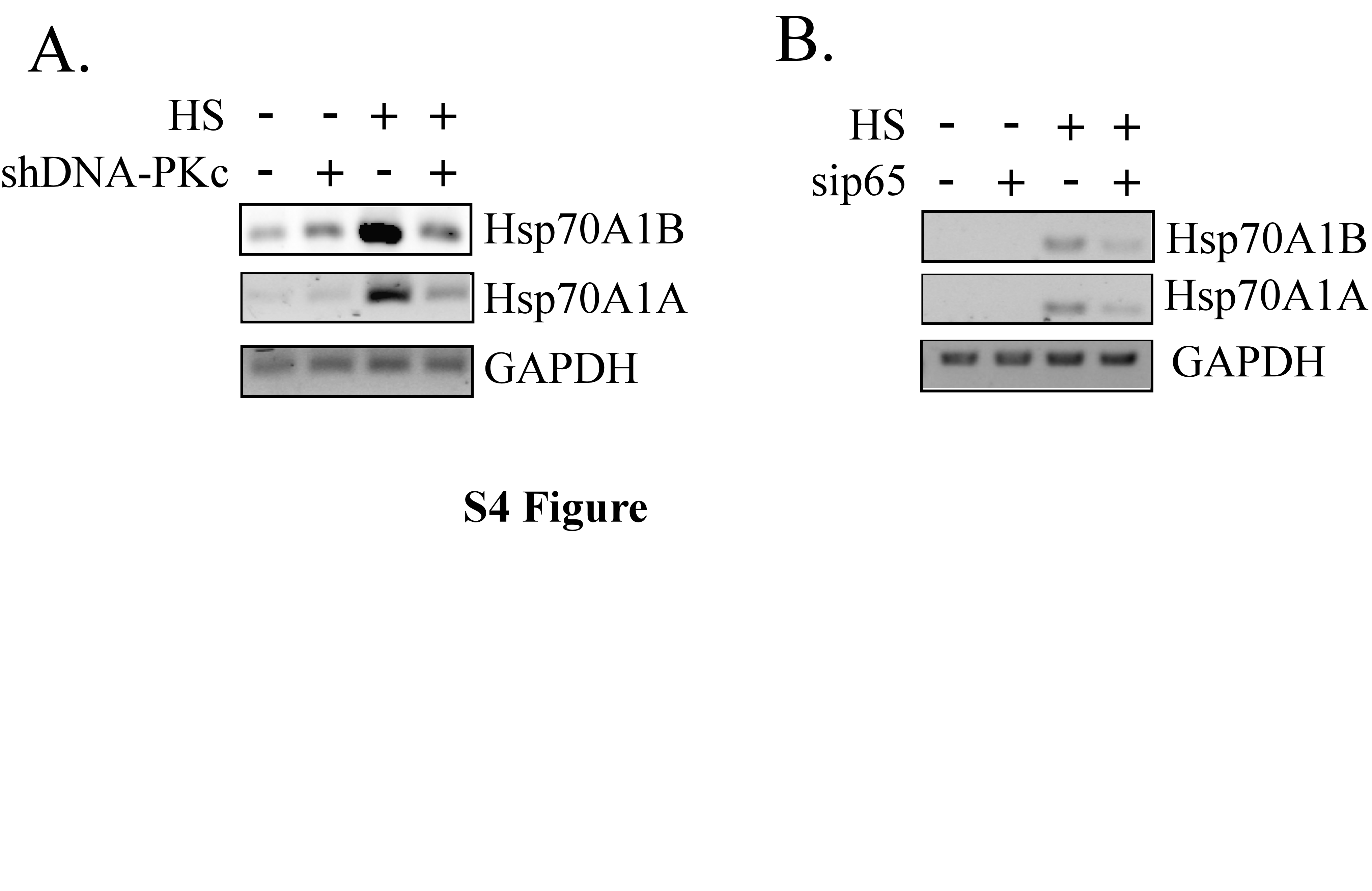

Supplement: S4 Fig — Representative ethidium bromide stained agarose gel indicating relative transcript levels in HeLa cells pre-treated with shDNA-PKc or sip65/ReLA following heat shock determined by RT-PCR assay. Panel (A) represents shDNA-PK pretreated samples. Panel (B) represents sip65/RelA pretreated samples. GAPDH levels were determined as an internal loading control. (TIF) [file pone.0168165.s004.tif]

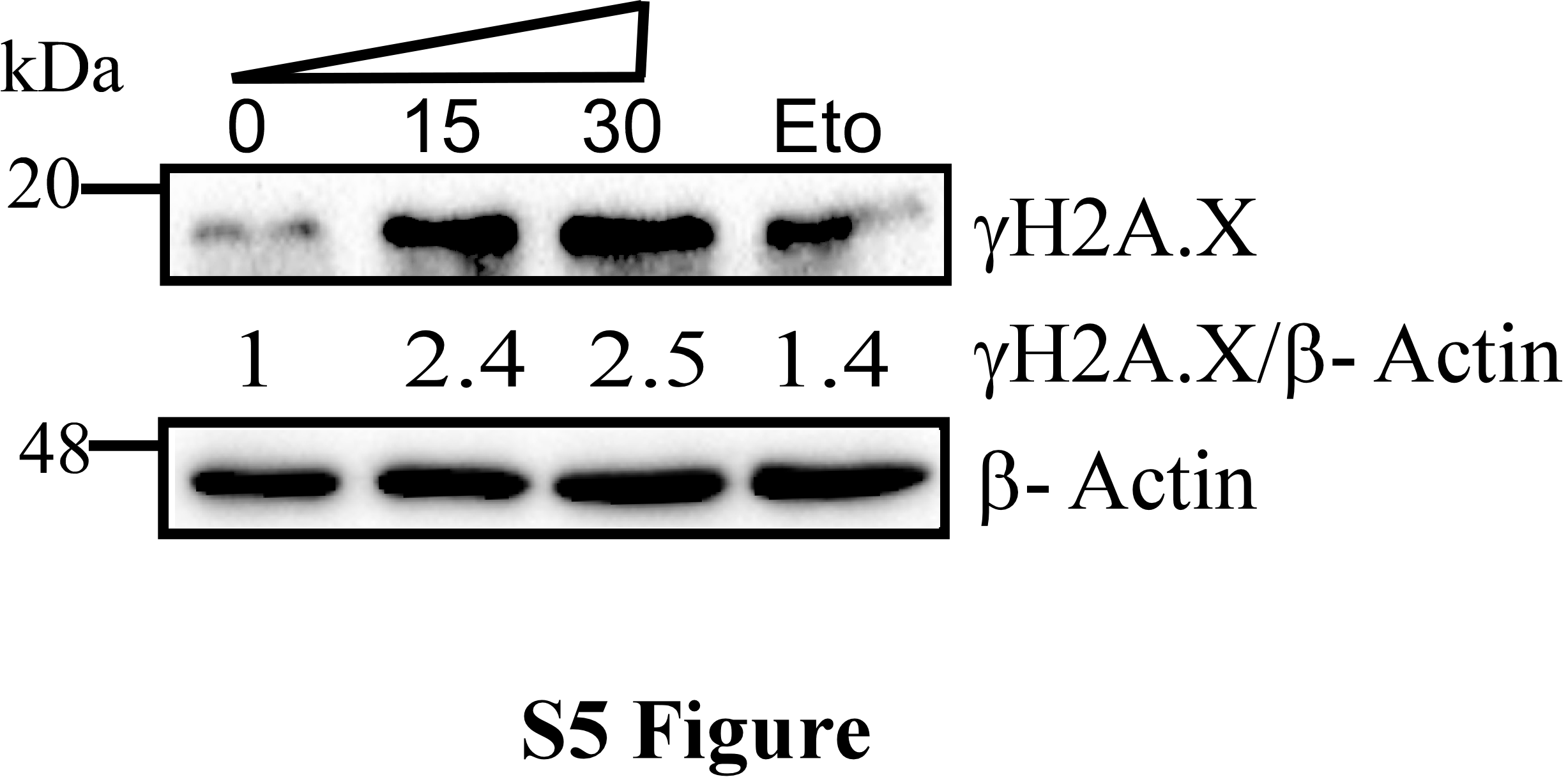

Supplement: S5 Fig — Immunoblot showing levels of phosphorylated γH2A.X protein in whole cell lysates isolated from HeLa cells that were subjected to different duration (0, 15, 30 min) of heat shock treatment or not. β-actin level was determined as internal loading control. (TIF) [file pone.0168165.s005.tif]
